# Supplementary material for: Cellular hierarchy insights reveal leukemic stem-like cells and early death risk in acute promyelocytic leukemia
Source: Nat Commun. 2024 Feb 16;15:1423. doi: 10.1038/s41467-024-45737-7 (PMC10873341; doi:10.1038/s41467-024-45737-7)
Supplement: Supplementary file 1 — Supplementary Information [file 41467_2024_45737_MOESM1_ESM.pdf]

## Supplementary Figures

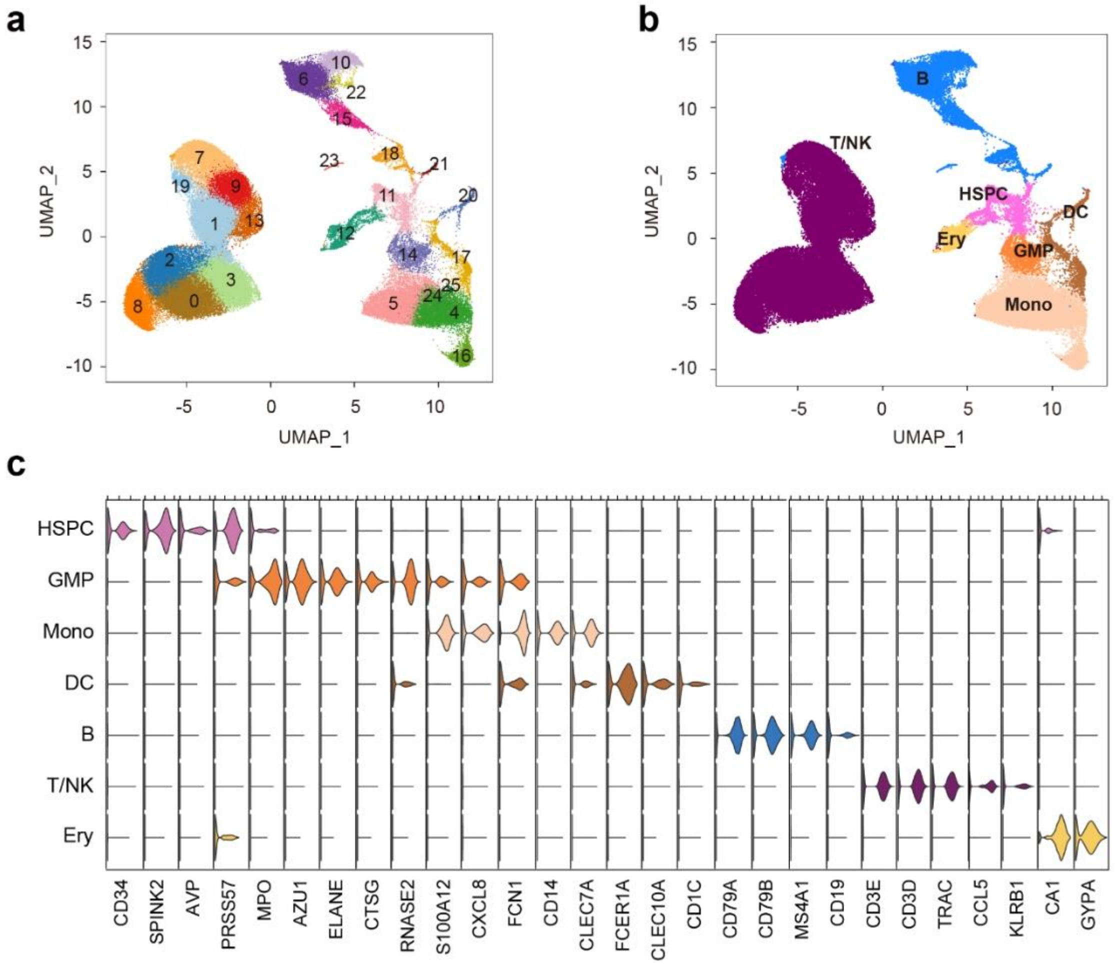


**Supplementary Fig. 1.** Cell type identification in normal BM samples. **a,b,** UMAP plots of normal BM cells (n = 102,919 cells), colored by cell clusters (**a**) and inferred cell types (**b**). **c,** Violin plot showing normalized expression levels and expression percentages of cell-type-specific genes in seven cell populations in normal BM cells.

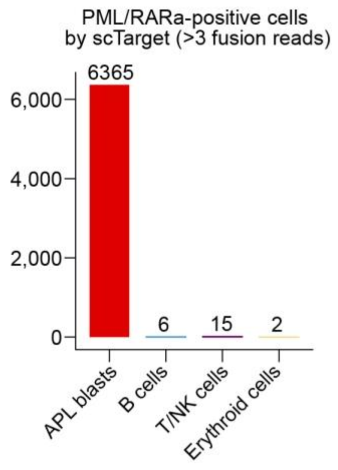


**Supplementary Fig. 2.** Comparison of the numbers of PML/RARα-positive cells detected by scTarget in APL blasts, B cells, T/NK cells, and erythroid cells from two APL patient samples. Cells detected with more than three PML/RARα fusion reads were calculated.

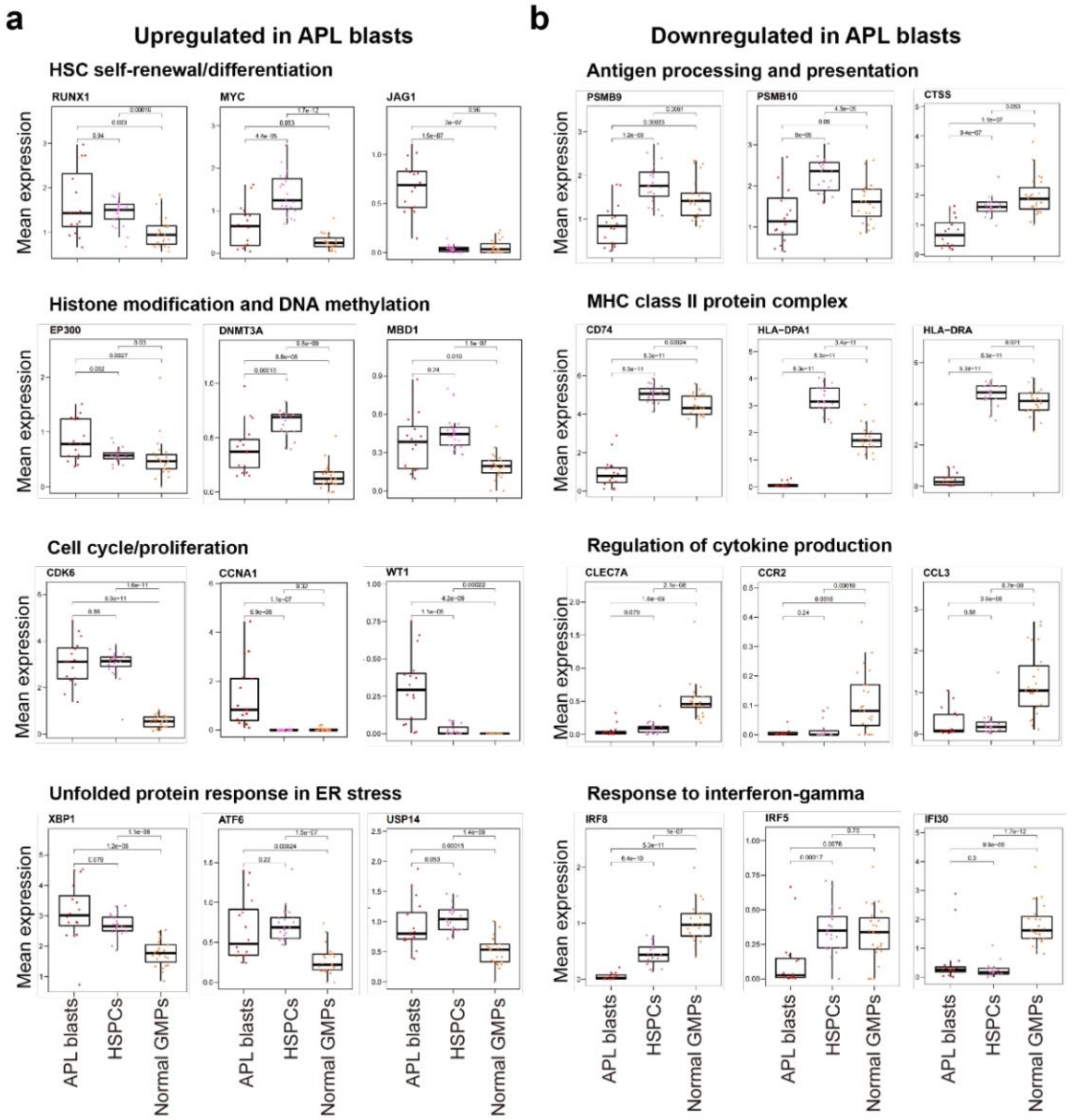


**Supplementary Fig. 3.** Box plots of selected DEGs upregulated (**a**) or downregulated (**b**) in APL blasts, respectively. The x-axes show APL blasts (n = 16 APL BM samples), HSPCs (n = 23 normal BM samples), and normal GMPs (n = 23 normal BM samples), and the y-axes represent the mean expression of the genes. The *P*-values were calculated using the Wilcoxon rank-sum test. Two-sided *P*-values were calculated. In the boxplot, a black line within the box marks the median. The bottom and top of the box are located at the 25$^{th}$ and 75$^{th}$ percentiles, respectively. The bars represent values more than 1.5 times the interquartile range from the border of each box.

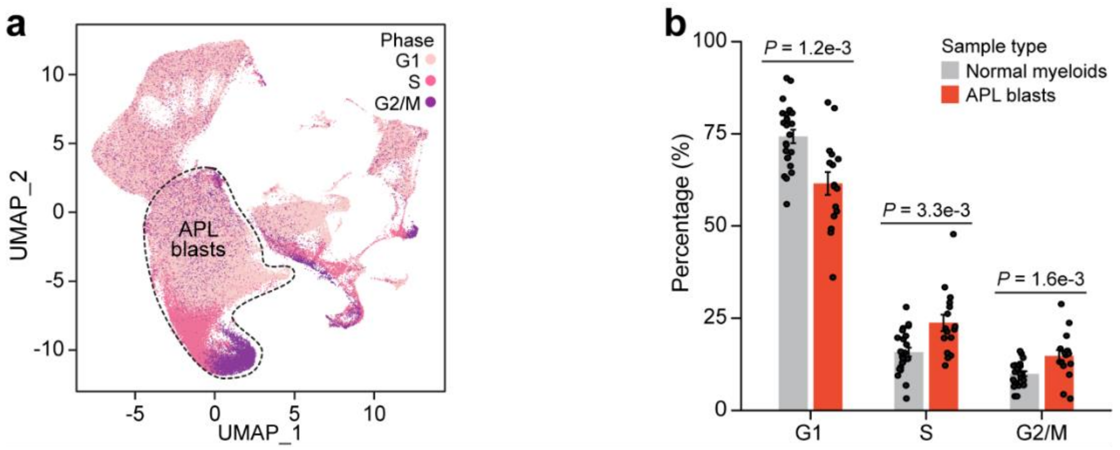


**Supplementary Fig. 4.** Cell cycle status analysis. **a**, UMAP plot of APL and normal BM cells (n = 239,332 cells), colored by different cell cycle phases (G1, S, and G2/M). **b**, Comparison of the cell cycle status between APL blasts and normal myeloid cells. The *P*-values were calculated using the Wilcoxon rank-sum test. Two-sided *P*-values were calculated.

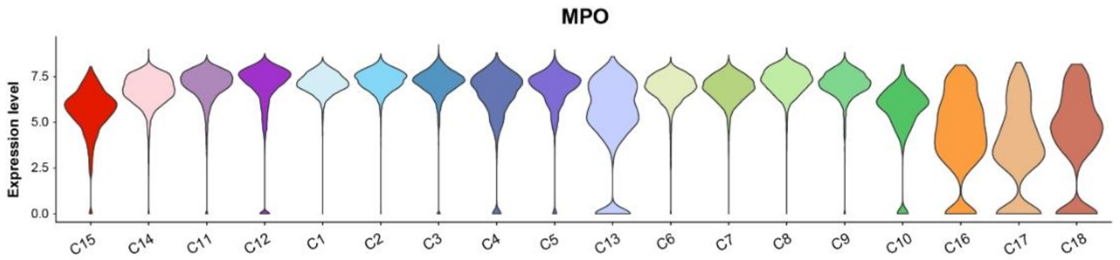


**Supplementary Fig. 5.** Expression levels of MPO in all 18 cell clusters of APL blasts (n = 126,802 cells).

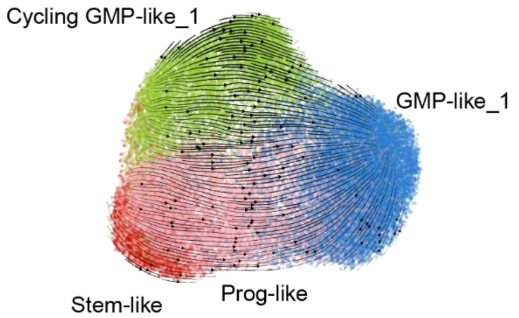


**Supplementary Fig. 6.** RNA velocity-based differentiation trajectory analysis of four main clusters (n = 38,403 cells), including Stem-like, Prog-like, Cycling GMP-like 1, and GMP-like 1. The analysis reveals that cycling GMP-like cells originate from primitive APL subsets and transition towards mature GMP-like subsets. This finding aligns with recent research emphasizing that cell cycle entry is an integral part of the continuum of cell differentiation.

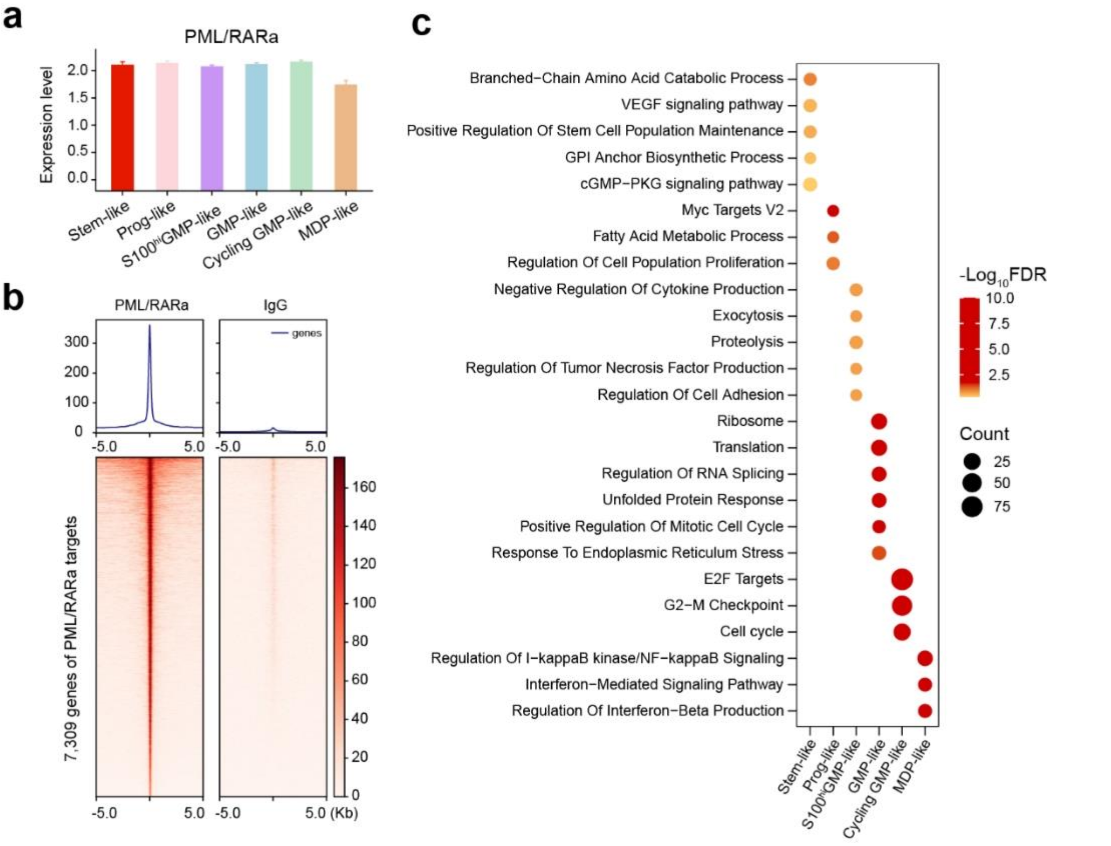


**Supplementary Fig. 7.** Branch-specific regulation of PML/RARα in the APL trajectory. **a**, Expression levels of PML/RARα across the 6 branches of APL blasts. Cells detected with more than three PML/RARα fusion reads were calculated (n = 6,365 cells). **b,** Density plot showing the enrichment of endogenous PML/RARα surrounding the summit of 7,309 PML/RARα peaks in NB4 cells, ranked by PML/RARα peak intensity. **c**, Pathways significantly enriched for PML/RARα-regulated branch-specific marker genes in different branches. The *P*-values were calculated by Enrichr (https://maayanlab.cloud/Enrichr/). Two-sided *P*-values were calculated.

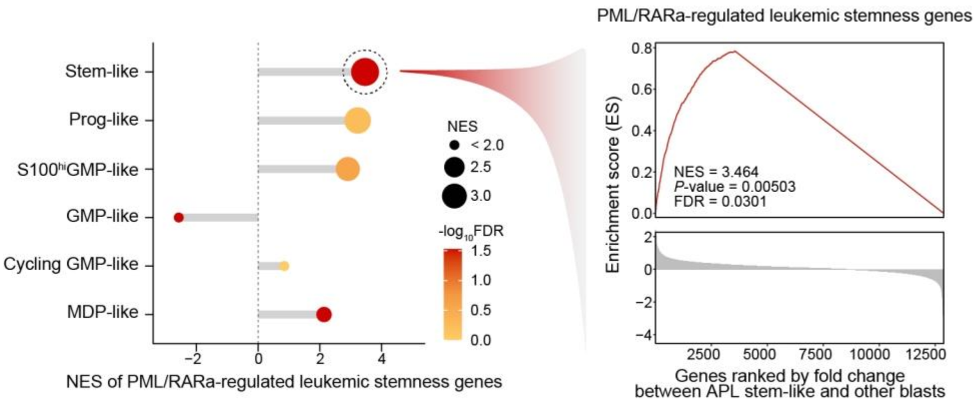


**Supplementary Fig. 8.** Enrichment analysis of PML/RARα-regulated leukemic stemness genes (n = 991 genes) illustrating the highest significant enrichment in APL stem-like cells. The left dot plot shows the NES obtained by comparing cells in each branch with other APL cell branches. The right panel shows a GSEA enrichment plot of PML/RARα-regulated leukemic stemness genes in the stem-like cells compared to the other APL cell branches. Genes were ranked based on the fold change between APL stem-like cells and other APL cell branches. The *P*-values were calculated by GSEA. Two-sided *P*-values were calculated.

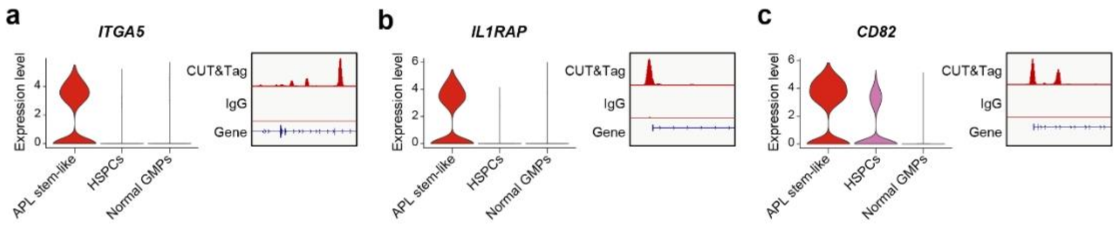


**Supplementary Fig. 9**. Expression levels of *ITGA5* (**a**, left panel), *IL1RAP* (**b**, left panel), and *CD82* (**c**, left panel) in APL stem-like cells, HSPCs, and normal GMPs, respectively. Genome browser tracks illustrate the PML/RARα binding (CUT&Tag-seq) to the promoters of *ITGA5* (**a**, right panel), *IL1RAP* (**b**, right panel), and *CD82* (**c**, right panel), respectively.

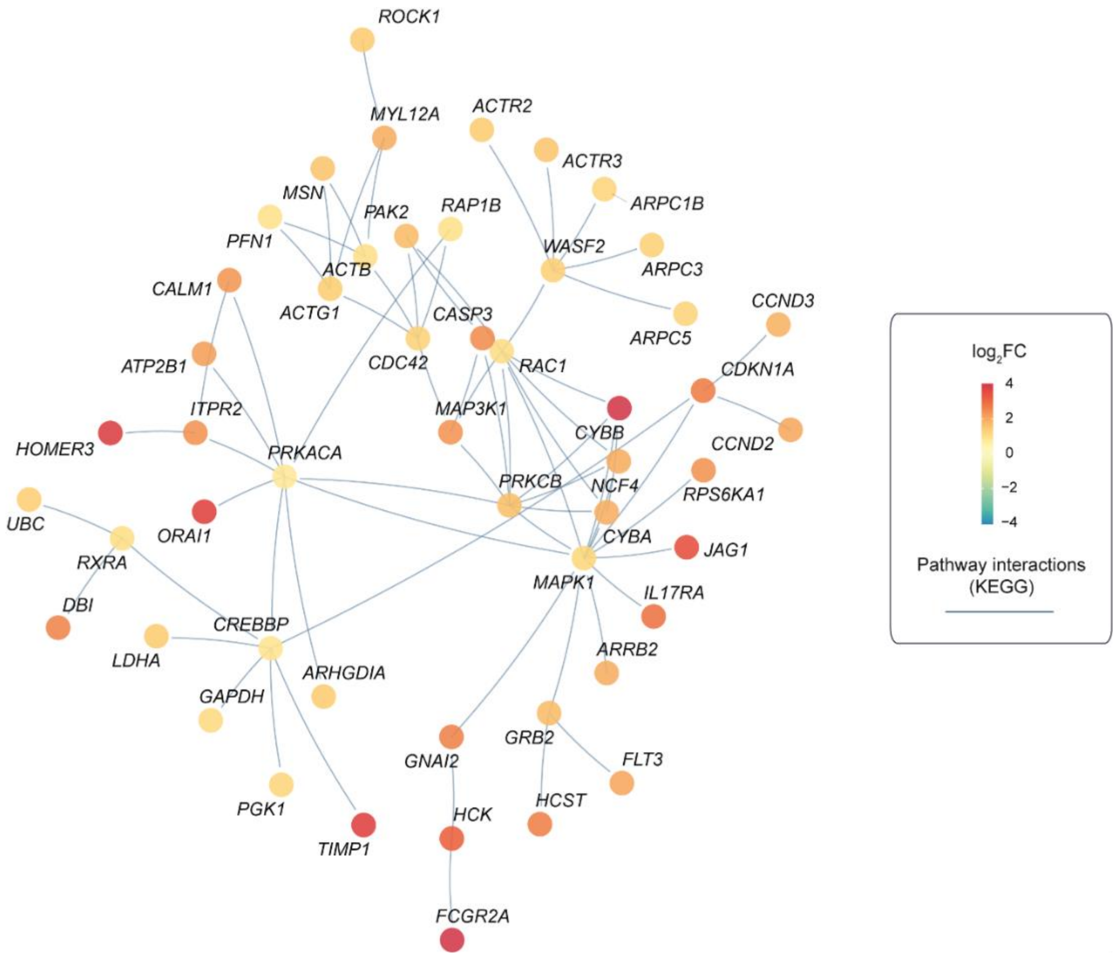


**Supplementary Fig. 10.** Crosstalk between LSC-associated pathways identified in APL stem-like cells. Nodes (PML/RARα targets) are color-coded by the $\log_2$(fold changes) of gene expression levels in APL stem-like cells compared to HSPCs. FC, fold changes. Notably, this 51-gene pathway crosstalk contains PML/RARα target genes that are uniformly upregulated in APL stem-like cells, including genes that encode well-known LSC markers and genes that are important for APL leukemogenesis.

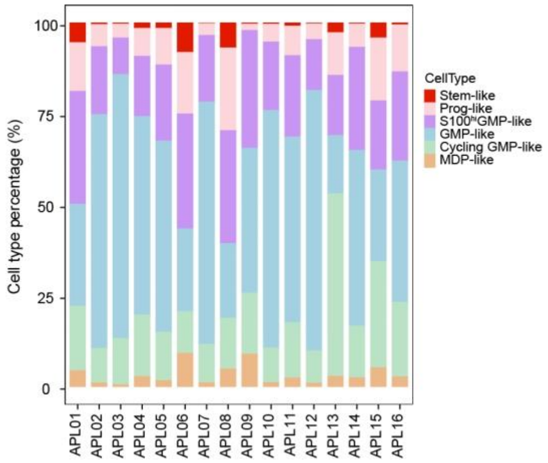


**Supplementary Fig. 11.** Proportions of the 6 cell branches for each APL patient.

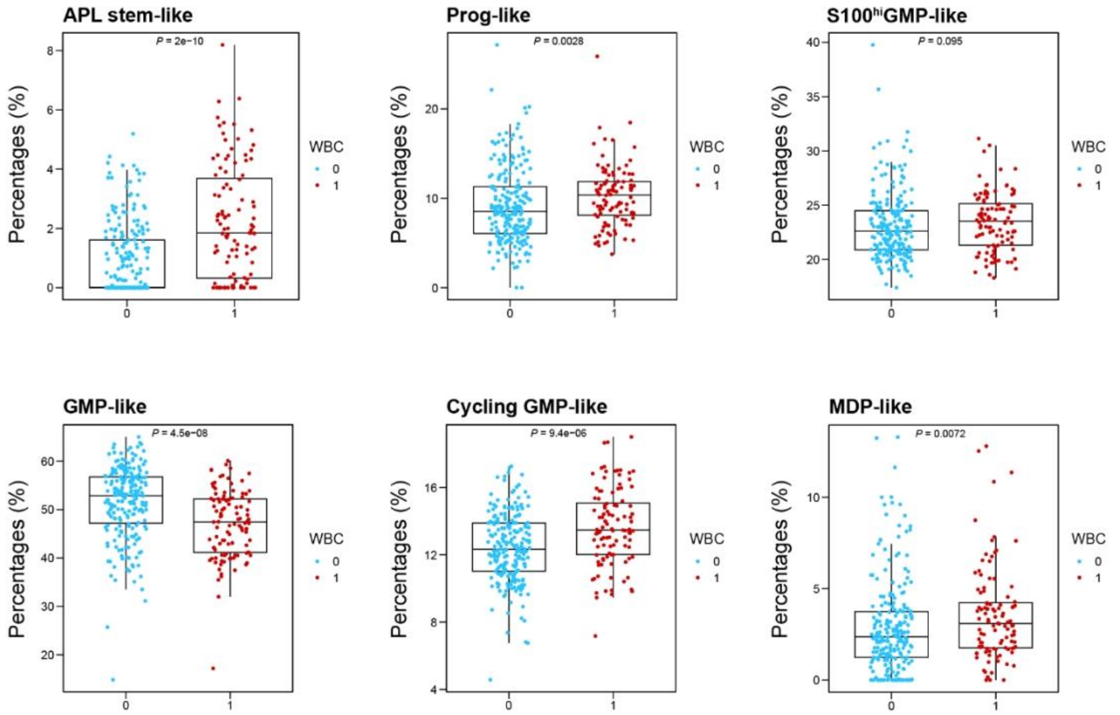


**Supplementary Fig. 12.** Correlation between the proportion of each leukemic cell type and the WBC counts in the 323 APL patients. 0, ≤ 10 × $10^9$/L; 1, > 10 × $10^9$/L. The *P*-values were calculated using the Wilcoxon rank-sum test. Two-sided *P*-values were calculated. In the boxplot, a black line within the box marks the median. The bottom and top of the box are located at the 25th and 75th percentiles, respectively. The bars represent values more than 1.5 times the interquartile range from the border of each box.

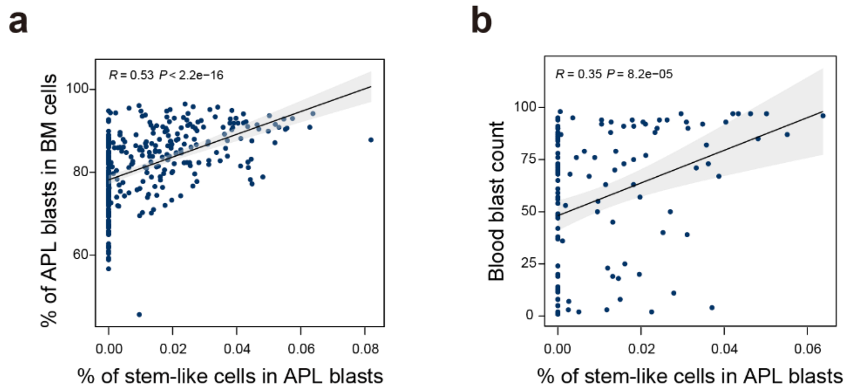


**Supplementary Fig. 13.** Correlation between the proportion of stem-like cells in APL blasts and the percentage of APL blasts in BM cells (**a**), or the blood blast count (**b**). The *P*-values were calculated using the Pearson's correlation. Two-sided *P*-values were calculated. **a**, 323 patients were included; **b**, 121 patients with blood blast count information were included.

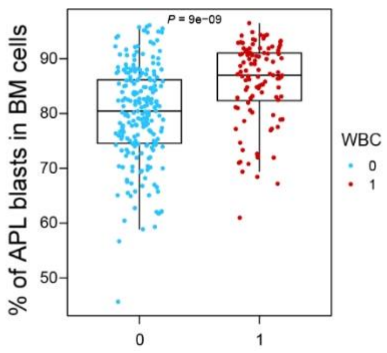


**Supplementary Fig. 14.** Correlation between the percentage of APL blasts in BM cells and the WBC counts in the 323 APL patients. 0, $\leq 10 \times 10^9$/L; 1, $> 10 \times 10^9$/L. The *P*-values were calculated using the Wilcoxon rank-sum test. Two-sided *P*-values were calculated. In the boxplot, a black line within the box marks the median. The bottom and top of the box are located at the 25$^{th}$ and 75$^{th}$ percentiles, respectively. The bars represent values more than 1.5 times the interquartile range from the border of each box.

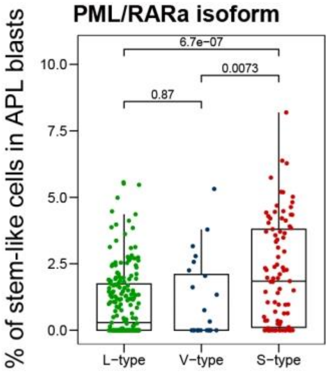


**Supplementary Fig. 15.** Correlation between the types of PML/RARα isoforms and the percentage of APL stem-like cells in the 323 APL patients. The L-, S-, and V-types represent the long, short, and variant isoforms of PML/RARα, respectively defined by the breakpoint of PML on intron 6, intron 3, and exon 6. The *P*-values were calculated using the Wilcoxon rank-sum test. Two-sided *P*-values were calculated. In the boxplot, a black line within the box marks the median. The bottom and top of the box are located at the 25$^{th}$ and 75$^{th}$ percentiles, respectively. The bars represent values more than 1.5 times the interquartile range from the border of each box.

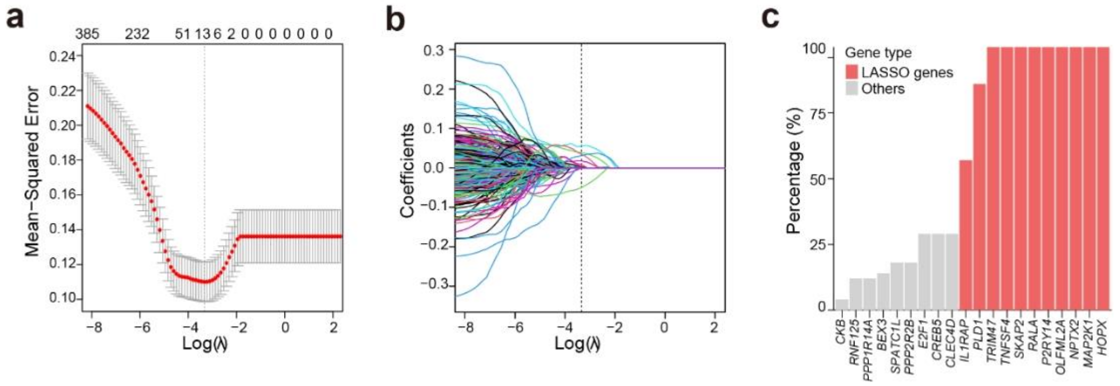


**Supplementary Fig. 16.** The selection criteria for the APL stemness score. **a**, Cross-validation in the LASSO model to select the tuning parameter. The X-axis represents the log (lambda, λ) value, and the Y-axis for the partial likelihood deviance. The minimum mean cross-validated error of λ is selected. The lower X-axis represents the lambda value, and the upper X-axis scale for the number of genes in the LASSO model. **b**, Coefficient selection and variable screening of LASSO. **c**, Occurrence ratio of LASSO genes after repeated 100 times using different random seeds.

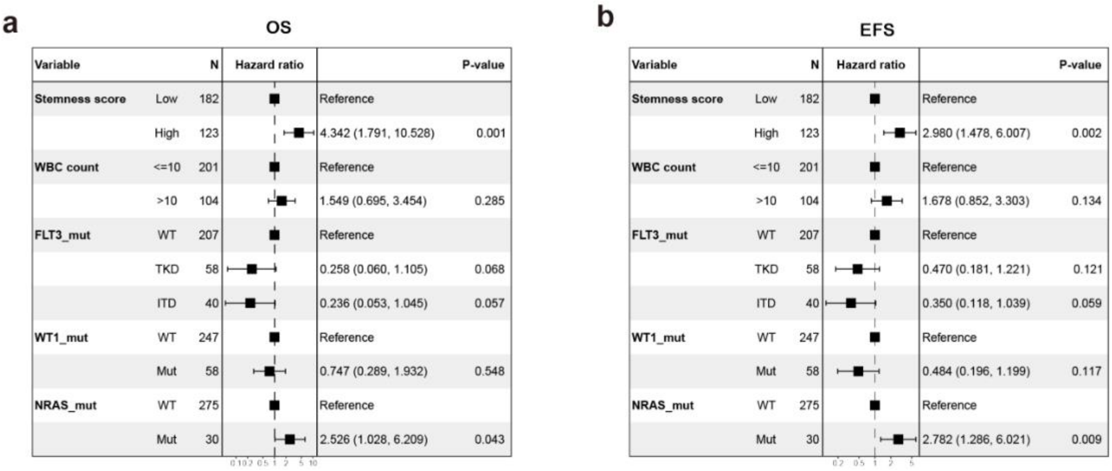


**Supplementary Fig. 17.** Forest plots of multivariate Cox analysis of risk factors for OS (**a**) and EFS (**b**) in APL patients (n = 305 patients with prognostic information). In the forest plot, the center represents the hazard ratio for each risk factor; the error bars represent two-sided 95% CIs (Cox regression multivariate analysis). N, the number of patients; WT, wild-type; mut, mutation.

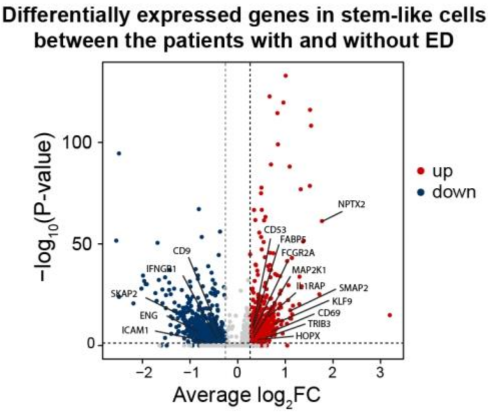


**Supplementary Fig. 18.** Volcano plot illustrating differentially expressed genes (n = 13,250 genes) in stem-like cells between patients with and without ED. Red dots represent upregulated genes, and blue dots represent downregulated genes. The *P*-values were calculated using the R package limma. Two-sided *P*-values were calculated.

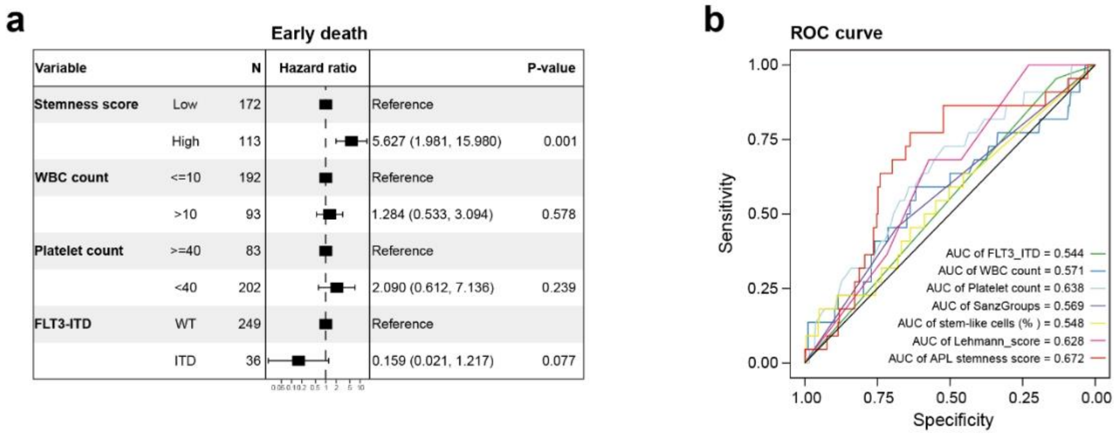


**Supplementary Fig. 19.** Comparison of the APL stemness score against known risk factors and established risk assessment tools for APL. **a,** Forest plot of multivariate Cox analysis illustrates the APL stemness score as an independent risk factor for predicting early death in APL patients. In the forest plot, the center represents the hazard ratio for each risk factor; the error bars represent two-sided 95% CIs (Cox regression multivariate analysis). WT, wild-type FLT3; N, the number of patients. **b,** Receiver Operating Characteristic (ROC) curve and the corresponding Area Under the Curve (AUC) values for evaluating the performance of the LASSO model, along with other models, genetic events, and clinical features in predicting early death. The analysis included 285 APL patients, among whom 22 experienced early death and 263 achieved complete remission.

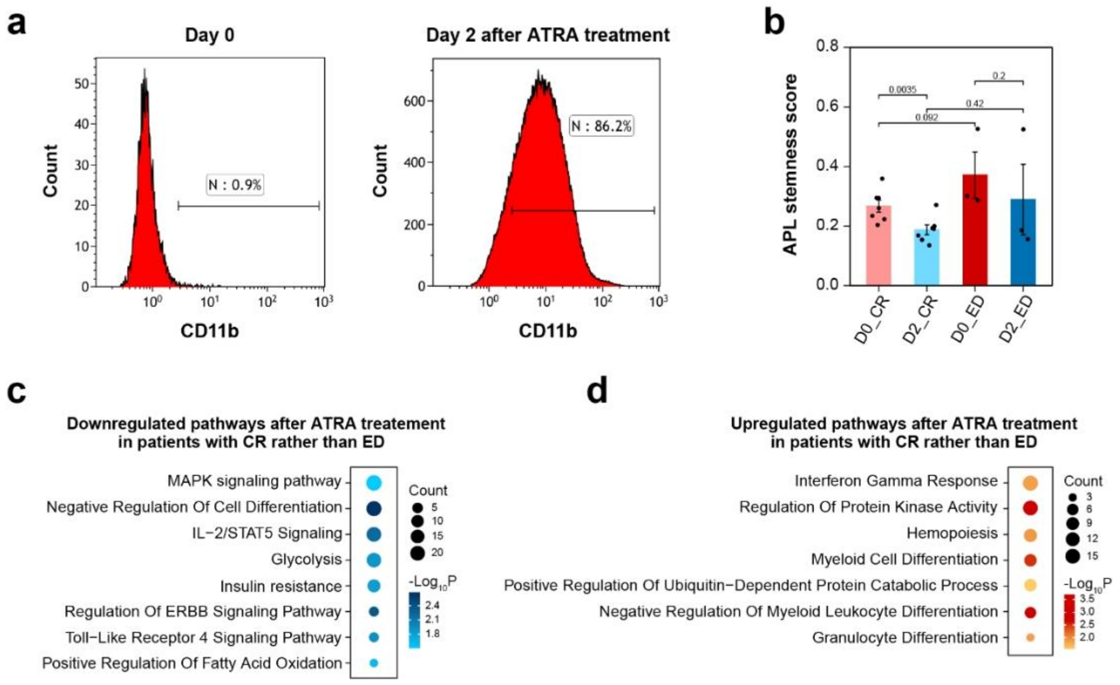


**Supplementary Fig. 20.** ATRA-induced changes after two days of treatment in APL patients. **a,** Comparison of CD11b expression before (Day 0, left panel) and after (Day 2, right panel) treatment with ATRA in the APL05 patient, as detected by FCM. **b**, Comparison of the APL stemness score between patients who achieved complete remission (CR; n = 7) and those who experienced early death (ED; n = 3) on Day 0 (D0) and Day 2 (D2) after ATRA treatment. The *P*-values were calculated using the Wilcoxon rank-sum test. One-sided *P*-values were calculated. **c,d,** Pathways significantly enriched for genes that were downregulated (**c**) or upregulated (**d**) post-ATRA treatment in patients who achieved CR but not in those who experienced ED. The *P*-values were calculated by Enrichr (https://maayanlab.cloud/Enrichr/). Two-sided *P*-values were calculated.

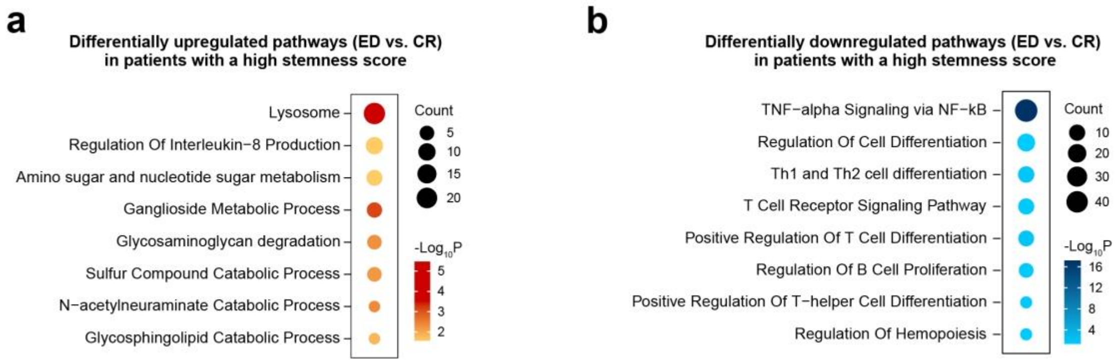


**Supplementary Fig. 21.** Differentially upregulated (**a**) and downregulated pathways (**b**) in patients who experienced early death (ED; n =3) compared to patients who achieved complete remission (CR; n =7) among patients with a higher stemness score (ED vs. CR). The *P*-values were calculated by Enrichr (https://maayanlab.cloud/Enrichr/). Two-sided *P*-values were calculated.

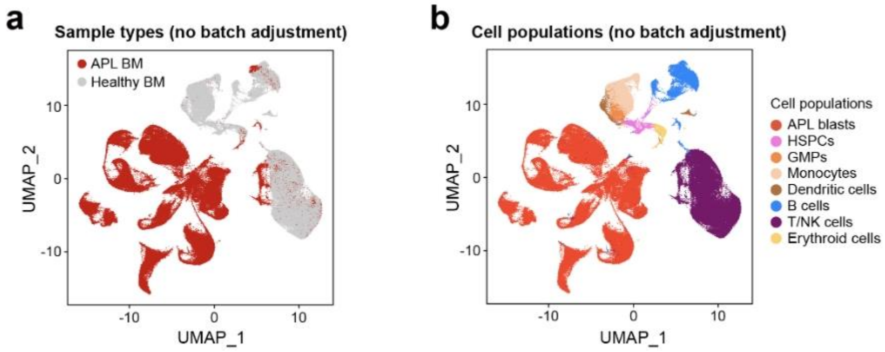


**Supplementary Fig. 22.** UMAP plots of APL and normal BM cells (n = 239,332 cells), colored by sample types (**a**) and inferred cell populations (**b**), respectively. No batch adjustment was performed.

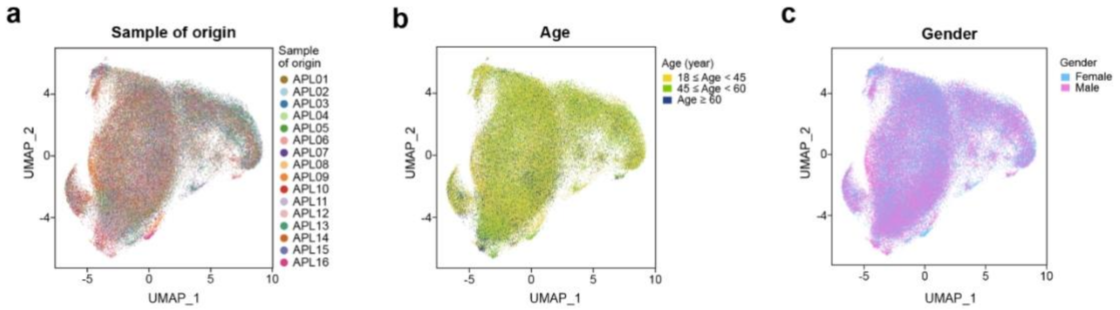


**Supplementary Fig. 23.** UMAP plots depicting APL blasts from the 16 APL patients after batch effect adjustment. Cells (n = 126,802 cells) are colored based on their respective sample origin (**a**), different age ranges (**b**), and genders (**c**), respectively. These results demonstrate that our identified clusters of APL blasts were not influenced by potential confounding variables such as age and gender.

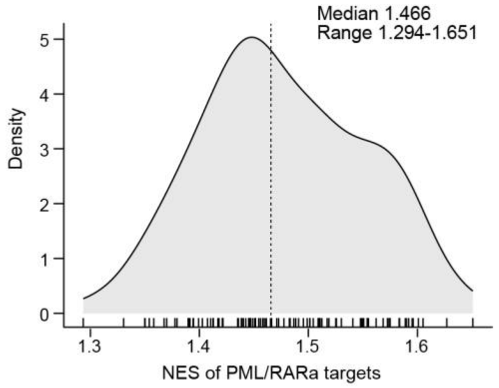


**Supplementary Fig. 24.** Distribution of NES calculated for PML/RARα targets between the APL stem-like population and normal HSPCs after 100 iterations. NES were calculated using GSEA. NES, normalized enrichment score. 500 genes are selected for each iteration.
