# Supplementary material for: Cellular hierarchy insights reveal leukemic stem-like cells and early death risk in acute promyelocytic leukemia
Source: Nat Commun. 2024 Feb 16;15:1423. doi: 10.1038/s41467-024-45737-7 (PMC10873341; doi:10.1038/s41467-024-45737-7)
Supplement: Supplementary file 3 — Description of Additional Supplementary Files [file 41467_2024_45737_MOESM3_ESM.pdf]

Description of Additional Supplementary Files

Supplementary Data 1. Patient clinical characteristics and sample information.

Supplementary Data 2. Cell type-characteristic genes in the UMAP representation of APL blasts.

Supplementary Data 3. Branch-characteristic genes in the UMAP representation of APL blasts.

Supplementary Data 4. Genome-wide binding sites of PML/RARα identified by CUT&Tag-seq.

Supplementary Data 5. PML/RARα-regulated branch-specific marker genes in different branches.

Supplementary Data 6. Differentially expressed genes (DEGs) between APL stem-like cells and normal HSPCs, along with the information on PML/RARα targets.

Supplementary Data 7. List of pathway crosstalk genes, along with their manually curated relatedness to stemness and APL biology.

Supplementary Data 8. Clinical and molecular features of 323 APL patients.

Supplementary Data 9. Proportions of the 6 cell branches in 16 APL patients.

Supplementary Data 10. Mean expression of genes in stem-like, Prog-like, S100^{hi}GMP-like, and GMP-like cell populations on Day 0 (D0) and Day 2 (D2) after ATRA therapy, respectively.

Supplementary Data 11. Log$_2$FC of gene expression in 9 APL patients by comparing bulk RNA-seq data before and after ATRA treatment for two days (Day 2 vs. Day 0), respectively.
