# Supplementary material for: Cellular hierarchy insights reveal leukemic stem-like cells and early death risk in acute promyelocytic leukemia
Source: Nat Commun. 2024 Feb 16;15:1423. doi: 10.1038/s41467-024-45737-7 (PMC10873341; doi:10.1038/s41467-024-45737-7)
Supplement: Supplementary file 15 — Reporting Summary [file 41467_2024_45737_MOESM15_ESM.pdf]

nature portfolio

Corresponding author(s): Kankan Wang

Last updated by author(s): Feb 2, 2024

Reporting Summary

Nature Portfolio wishes to improve the reproducibility of the work that we publish. This form provides structure for consistency and transparency in reporting. For further information on Nature Portfolio policies, see our Editorial Policies and the Editorial Policy Checklist.

Statistics

For all statistical analyses, confirm that the following items are present in the figure legend, table legend, main text, or Methods section.

n/a	Confirmed	
☐	☒	The exact sample size (*n*) for each experimental group/condition, given as a discrete number and unit of measurement
☐	☒	A statement on whether measurements were taken from distinct samples or whether the same sample was measured repeatedly
☐	☒	The statistical test(s) used AND whether they are one- or two-sided<br>*Only common tests should be described solely by name; describe more complex techniques in the Methods section.*
☒	☐	A description of all covariates tested
☐	☒	A description of any assumptions or corrections, such as tests of normality and adjustment for multiple comparisons
☐	☒	A full description of the statistical parameters including central tendency (e.g. means) or other basic estimates (e.g. regression coefficient) AND variation (e.g. standard deviation) or associated estimates of uncertainty (e.g. confidence intervals)
☐	☒	For null hypothesis testing, the test statistic (e.g. *F*, *t*, *r*) with confidence intervals, effect sizes, degrees of freedom and *P* value noted<br>*Give P values as exact values whenever suitable.*
☒	☐	For Bayesian analysis, information on the choice of priors and Markov chain Monte Carlo settings
☐	☒	For hierarchical and complex designs, identification of the appropriate level for tests and full reporting of outcomes
☐	☒	Estimates of effect sizes (e.g. Cohen's *d*, Pearson's *r*), indicating how they were calculated

Our web collection on statistics for biologists contains articles on many of the points above.

Software and code

Policy information about availability of computer code

Data collection	The codes used in the paper are available on GitHub (https://nrctm-bioinfo.github.io/APL_stemness) and Zenodo (https://doi.org/10.5281/zenodo.10437695). Source data are provided as a Source Data file within the paper, also accessible through the project-dedicated website 'TACH' (http://www.genetictargets.com/tach).
Data analysis	Software including SRA-toolkit (version 2.11.0), cellranger (version 6.0.2), Monocle2 (version 2.24.0), Salmon (version 1.5.1), e1071 (version 1.7.13), MSigDB (version 7.4), XGR (version 1.1.4), Seurat (version 4.3.0), Harmony (version 1.0.3), enrichR (version 3.2), VIPER (version 1.26.0), DoRothEA (version 1.4.2), BiocNeighbors (version 1.10.0).

For manuscripts utilizing custom algorithms or software that are central to the research but not yet described in published literature, software must be made available to editors and reviewers. We strongly encourage code deposition in a community repository (e.g. GitHub). See the Nature Portfolio guidelines for submitting code & software for further information.

Data

Policy information about availability of data

All manuscripts must include a data availability statement. This statement should provide the following information, where applicable:

- Accession codes, unique identifiers, or web links for publicly available datasets
- A description of any restrictions on data availability
- For clinical datasets or third party data, please ensure that the statement adheres to our policy

The raw sequencing data reported in this paper have been deposited in the Genome Sequence Archive in National Genomics Data Center, China National Center for Bioinformation/Beijing Institute of Genomics, Chinese Academy of Sciences (https://ngdc.cncb.ac.cn/gsa-human). These data are accessible under the accession number 'GSA-Human: HRA003777' (https://ngdc.cncb.ac.cn/gsa-human/browse/HRA003777). These data are under controlled access by human privacy regulations and are only available for research purposes. Access to the data can be granted following approval from the Data Access Committee of the GSA-human database, as detailed at https://ngdc.cncb.ac.cn/gsa-human/document/GSA-Human_Request_Guide_for_Users_us.pdf. Data are accessible to researchers who meet the criteria for access as defined by the GSA-human database guidelines. Access requests are usually processed within approximately four weeks, and data will be available for three months once access is granted. All sequencing data, including scRNA-seq and bulk RNA-seq data, are also available in NODE under the accession number OEP003829 (https://www.biosino.org/node/project/detail/OEP003829). The public datasets utilized in this study are available in the GEO database. These include scRNA-seq data from 23 healthy BM samples (accession code GSE120221 and GSE130116), the APL cohort data (GSE172057), and the PML/RARα target data generated from CUT&Tag (GSE195776). Source data are provided as a Source Data file within the paper, also accessible through the project-dedicated website 'TACH' (http://www.genetictargets.com/tach). The remaining data are available within the Article, Supplementary Information or Source Data file.

Research involving human participants, their data, or biological material

Policy information about studies with human participants or human data. See also policy information about sex, gender (identity/presentation), and sexual orientation and race, ethnicity and racism.

Reporting on sex and gender	Detailed information about the patient's gender is provided in Supplementary Data 1. In our prospective study, patient selection was not based on gender, but our analysis indicated that gender distribution did not influence the identified clusters of APL blasts.
Reporting on race, ethnicity, or other socially relevant groupings	NA
Population characteristics	The APL bone marrow cells from 16 patients were all collected at diagnosis. Detailed characteristics were in Supplementary Data 1.
Recruitment	APL patients were randomly recruited in the clinical setting and we also confirmed that the patients in each analysis were representative of the broader APL patient demographic. The selection/recruitment was also contingent upon the availability of high-quality samples, which is a prerequisite for the reliable scRNA-seq analysis that our study relies on.
Ethics oversight	Written informed consent was obtained from the patient allowing for the publication of clinical information, and ethical approval was obtained from the Ethics Committees of Ruijin Hospital, Shanghai Jiao Tong University School of Medicine (2021/154).

Note that full information on the approval of the study protocol must also be provided in the manuscript.

Field-specific reporting

Please select the one below that is the best fit for your research. If you are not sure, read the appropriate sections before making your selection.

☒ Life sciences ☐ Behavioural & social sciences ☐ Ecological, evolutionary & environmental sciences

For a reference copy of the document with all sections, see nature.com/documents/nr-reporting-summary-flat.pdf

Life sciences study design

All studies must disclose on these points even when the disclosure is negative.

Sample size	This study included 16 de novo APL patients with the high-quality samples available.
Data exclusions	No data was excluded from the analysis.
Replication	The investigation of APL heterogeneity was achieved through the integrated analysis of scRNA-seq data from 16 de novo APL samples, and replication was not possible.
Randomization	There was no group allocation.
Blinding	There was no group allocation.

Reporting for specific materials, systems and methods

We require information from authors about some types of materials, experimental systems and methods used in many studies. Here, indicate whether each material, system or method listed is relevant to your study. If you are not sure if a list item applies to your research, read the appropriate section before selecting a response.

Materials & experimental systems

n/a	Involved in the study
☒	☐ Antibodies
☒	☐ Eukaryotic cell lines
☒	☐ Palaeontology and archaeology
☒	☐ Animals and other organisms
☒	☐ Clinical data
☒	☐ Dual use research of concern
☒	☐ Plants

Methods

n/a	Involved in the study
☒	☐ ChIP-seq
☒	☐ Flow cytometry
☒	☐ MRI-based neuroimaging
